# Coronary-Pulmonary Artery Fistula Recanalization on Coronary Computed Tomography Angiography Images

**DOI:** 10.3390/diagnostics11101921

**Published:** 2021-10-17

**Authors:** Paweł Gać, Adrian Martuszewski, Patrycja Paluszkiewicz, Rafał Poręba

**Affiliations:** 1 Centre for Diagnostic Imaging, 4th Military Hospital, Weigla 5, PL 50-981 Wroclaw, Poland; 2 Department of Population Health, Division of Environmental Health and Occupational Medicine, Wroclaw Medical University, Mikulicza-Radeckiego 7, PL 50-368 Wroclaw, Poland; adrian.martuszewski@student.umw.edu.pl; 3 Department of Emergency Medical Service, Wroclaw Medical University, Bartla 5, PL 50-367 Wroclaw, Poland; patrycja.paluszkiewicz@student.umw.edu.pl; 4 Department of Internal and Occupational Diseases, Hypertension and Clinical Oncology, Wroclaw Medical University, Borowska 213, PL 50-556 Wroclaw, Poland; rafal.poreba@umw.edu.pl

**Keywords:** computed tomography angiography, coronary-pulmonary artery fistula, fistula recanalization

## Abstract

Coronary computed tomography angiography (CCTA) is a non-invasive diagnostic method used (apart from the diagnosis of coronary artery disease) in the diagnosis of malformations of the coronary circulation and monitoring the effects of their treatment. In this paper, the authors present the case of recanalization of the coronary-pulmonary fistula, which was surgically closed in the past. This case highlights that follow-up CCTA after surgical treatment of coronary artery fistula should be performed in every patient. The recommendations regarding the frequency of such follow-up should be made.

A 74-year-old woman came to the cardiology outpatient clinic due to deterioration of exercise tolerance. Her medical history included surgical closure of the fistula between the pulmonary artery and the left anterior descending (LAD) at the age of 45 years. Coronary angiography performed at that time showed no other coronary artery lesions.

The patient had a history of cardiac arrhythmias other than atrial fibrillation, ventricular arrhythmia, mild bicuspid regurgitation, hypercholesterolemia. In her medical history there was no hypertension, diabetes mellitus, stroke, acute coronary syndrome (ACS) or nicotinism. The woman suffered from dizziness, intermittent palpitations and discomfort in the precordial region while resting in the supine position. Heart palpitations occurred periodically during activity. The patient complained of intermittent pruritus in the lower limbs around the ankles, at the end of the day. Several weeks earlier, the patient was diagnosed with right lower leg varicose veins treated subcutaneously with low molecular weight heparin. She had degenerative changes of the osteoarticular system, especially of the spine. Blood pressure was normal, approximately 120/70 mmHg. The woman had a normal body mass index. The patient was treated with metoprolol (50 mg per day), betahistine (2 × 24 mg per day), diosmin (2 × 600 mg per day), vinpocetine (2 × 10 mg per day), acetylsalicylic acid (70 mg per day).

On echocardiography, left ventricular ejection fraction (LVEF) was 60%. The left ventricle was not enlarged, normal LV systolic function and impaired LV relaxation were observed. The ascending aorta was dilated. On ultrasonography of the carotid and vertebral arteries, the flows were normal. Small atherosclerotic plaques were present in the right internal carotid artery (RICA) and left internal carotid artery (LICA), but without hemodynamic significance.

Due to the patient’s medical history, evaluation of her current cardiovascular health and complaints, the patient was referred to the computed tomography laboratory for coronary computed tomography angiography (CCTA).

The CCTA examination showed the developmental anomaly of the left coronary artery course (Figure 1A–C). The left main (LM) was wide (up to approx. 0.7 cm in diameter). The proximal segment of the left anterior descending artery (LAD), to the level of the broad septal branch, was wide (diameter up to about 0.6 cm). The rest of the LAD was of typical width. A muscle bridge was observed in the middle segment of the LAD (Figure 1D). The first diagonal branch (Dg1) followed a typical course (Figure 1E). Approximately 1.8–2.0 cm below the LM division, a strong, wide (initially 0.4–0.5 cm in diameter) branch was shown, which branched from the LAD to the interventricular septum. This atypical branch after about 0.8–1.0 cm divides into a typical wide second-order branch with a further course typical for the septal branch and a wide (diameter up to about 0.5 cm) epicardial branch. After another about 0.9–1.1 cm, this epicardial branch subdivides into a typical second diagonal branch (Dg2) with a typical Dg2 topography and tortuous coronary artery fistula (CAF) (Figure 1F). Left circumflex artery (LCx) (Figure 1G), obtuse marginal branches (OM) and right coronary artery (RCA) followed the usual course. The branch OM1 from LCx was visualized just behind the LM division (Figure 1H). RCA was dominant (Figure 1I). The coronary arteries showed small, parietal, calcified atherosclerotic plaques that did not cause significant stenosis. There were numerous tortuous branches of a coronary artery fistula of varying width around the main pulmonary artery (MPA) (Figure 1J). The connection of one branch of the fistula with the MPA was visualized (Figure 1K). Thus, the diagnosis as a coronary-pulmonary artery fistula (CPAF) was clarified. In CPAF vessel topography, a high-density structure was visualized (Figure 1L). This structure may be the material that has been used to close the fistula during a previous operation, presumably a vascular coil. In the functional CCTA assessment, left ventricular ejection fraction was 65% (Figure 1M).

The pathological changes that were visualized in the CCTA (contrasted, numerous, small vessels of the coronary-pulmonary fistula) indicate the final diagnosis of recanalization of the coronary-pulmonary fistula, which was surgically closed in the past). The patient was referred to a cardiac surgery clinic for further treatment planning.

## Figures and Tables

**Figure 1:**
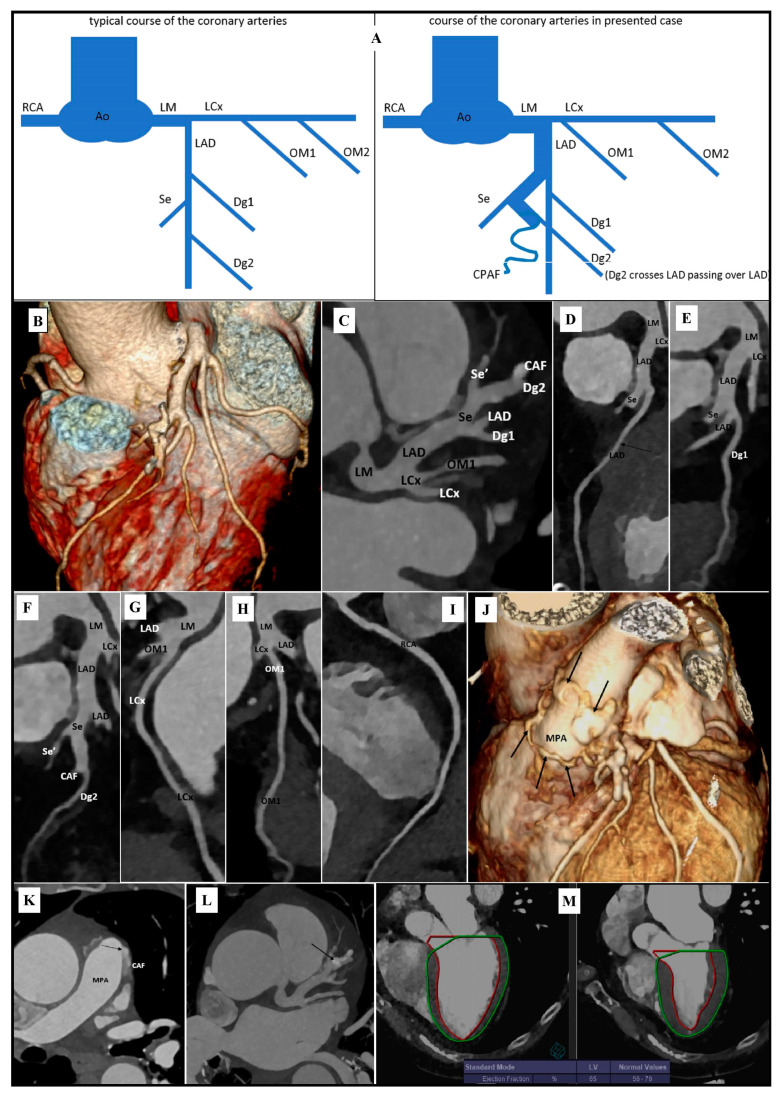
Recanalization of the coronary-pulmonary fistula in coronary artery computed tomography angiography: (**A**) Diagram of the course of the coronary arteries, which is typical; and observed in our case. (**B**) Volume Rendering Technique (VRT). Developmental anomaly of the left coronary artery course. (**C**) Maximum intensity projection (MIP). Axial view. Developmental anomaly of the left coronary artery course. (**D**) Curved planar reformation (CPR). Left anterior descending artery (LAD). Muscle bridge is marked with an arrow. (**E**) Curved planar reformation (CPR). 1st diagonal branch (Dg1). (**F**) Curved planar reformation (CPR). 2nd diagonal branch (Dg2). (**G**) Curved planar reformation (CPR). Left circumflex artery (LCx). (**H**) Curved planar reformation (CPR). 1st obtuse marginal branch (OM1). (**I**) Curved planar reformation (CPR). Right coronary artery (RCA). (**J**) Volume Rendering Technique (VRT). Branches of a coronary artery fistula around main pulmonary artery (MPA). Branches of the coronary artery fistula are marked with arrows. (**K**) Maximum intensity projection (MIP). Axial view. Coronary artery fistula (CAF) connection with main pulmonary artery (MPA). Connection is marked with an arrow. (**L**) Maximum intensity projection (MIP). Axial view. Postoperative changes after closure of the coronary fistula. High-density structure in coronary artery fistula is marked with an arrow. (**M**) Left ventricular functional assessment. Left ventricular ejection fraction (EF)-65%.

